# A twin-driven analysis on early aging biomarkers and associations with sitting-time and physical activity

**DOI:** 10.1371/journal.pone.0308660

**Published:** 2024-09-11

**Authors:** Ryan Bruellman, Shandell Pahlen, Jarrod M. Ellingson, Robin P. Corley, Sally J. Wadsworth, Chandra A. Reynolds

**Affiliations:** 1 Department of Genetics, Genomics and Bioinformatics, University of California Riverside, Riverside, California, United States of America; 2 Department of Psychology, University of California Riverside, Riverside, California, United States of America; 3 Institute for Behavioral Genetics, University of Colorado, Boulder, Colorado, United States of America; 4 Department of Psychiatry, Anschutz Medical Campus, University of Colorado, Aurora, Colorado, United States of America; 5 Department of Psychology and Neuroscience, University of Colorado, Boulder, Colorado, United States of America; Bangladesh University of Health Sciences, BANGLADESH

## Abstract

**Background:**

Current physical activity guidelines may be insufficient to address health consequences in a world increasing in sedentary behavior. Physical activity is a key lifestyle factor to promote healthy aging, but few studies examine activity in conjunction with sitting. We examine how activity intensity and sitting behavior influence health and the extent to which physical activity might counter sitting.

**Methods:**

We analyzed data from the Colorado Adoption/Twin Study of Lifespan behavioral development and cognitive aging (CATSLife) in adults aged 28–49 years (M = 33.16, SD = 4.93). We fit a linear mixed-effect model for body mass index (BMI) and total cholesterol/high-density lipoprotein ratio (TC/HDL). Leveraging the co-twin control approach, we explore the trade-off between sitting and physical activity.

**Results:**

Across established adulthood, TC/HDL and BMI demonstrated increasing age trends with prolonged sitting and vigorous activity inversely associated. Moreover, after considering sitting time, we found an age-equivalent benefit of vigorous exercise where those performing 30 minutes daily had expected TC/HDL and BMI estimates that mirrored sedentary individuals 5 and 10 years younger, respectively. Co-twin control analysis suggests partial exposure effects for TC/HDL, indicating greater vigorous activity may counter sitting-health effects but with diminishing returns.

**Conclusions:**

Our findings support the counteracting influence of prolonged sitting and physical activity on indicators of cardiovascular and metabolic health. A compensating role of vigorous activity on sitting health links is indicated while reducing sitting time appears paramount. Public health initiatives should consider sitting and vigorous activity in tandem in guidelines to promote health maintenance and combat accelerated aging.

## Introduction

Sitting for most of one’s waking hours is a commonality of life in developed countries, owing in part to transformed workplaces and the expansion of technology in our daily lives. Recent events such as the COVID-19 pandemic exacerbated sedentary behavior, with many US adults being classified as sedentary sitting over 8 hours per day [1, 2]. A sedentary lifestyle has been linked to negative health effects, particularly cardiovascular and metabolic health, and may increase mortality risk to a similar degree as obesity and smoking [3–5]. However, the comparison of the severity between sitting and smoking has been contested [6]. Positive correlations of sedentary behavior through prolonged sitting with indicators of obesity and dyslipidemia (i.e. high cholesterol) illustrate the importance of understanding prolonged sitting behavior; particularly how it may relate to aging via cardiovascular and metabolic disease susceptibility and progression [7]. This study offers a unique approach including the use of a co-twin control to better understand and manage confounds, allowing a better understanding of the impacts of sedentary behavior and how physical activity can potentially offset these impacts for premature aging.

Optimal cardiovascular and metabolic health are important components of healthy aging and offer significant public health implications. Aging-related cardiovascular diseases such as heart disease, stroke, and high blood pressure consistently remain among the top causes of age-adjusted death in the United States [8]. Total cholesterol (TC) levels and high-density lipoprotein (HDL) can offer insight into one’s cardiovascular health and the risks of various cardiovascular diseases. TC is comprised of HDL, low-density lipoprotein (LDL) levels, and triglyceride (TG); $TC=HDL+LDL+(\frac{1}{5})TG$ [9]. HDL is distinct from TG and LDL, in that it may prevent cardiovascular disease by promoting the reverse cholesterol transport process, preventing plaque buildup throughout the body which is commonly seen in those with high cholesterol at risk of negative cardiovascular events [10]. Thus, a full picture of the lipid profile should consider both HDL and TC.

Previous literature has indicated trends with higher sitting time positively correlating with LDL and TG levels (therefore positively associated with TC) [4]. While both TC and HDL are indicators used for determining risk for adverse cardiovascular events, taking the TC to HDL ratio (TC/HDL), also known as the Cardiac Risk Ratio offers a stronger indicator of various cardiovascular disease onset risks compared to considering TC and HDL levels separately [9, 11]. TC/HDL is classified by disease onset risk as either high (>5.0 for males, >4.4 for females), moderate (3.5–5.0 for males, 3.0–4.4 for females), or optimal (<3.5 for males, <3.0 for females) and the classifications do not change with age [12]. Previous studies have linked high TC/HDL to higher risk and incidence of aging-related adverse events such as stroke, metabolic syndrome, and heart disease in middle-aged and older adults [11, 13]. While TC/HDL changes do occur, outside of taking medications, improving one’s ratio often takes long-term lifestyle changes that can take several years to fully manifest [14]. Biomarkers such as TC/HDL highlight an important health indicator, that if maintained within recommended levels across early adulthood, may help to minimize later risk for disease susceptibility [12]. Understanding the links between sitting and physical activity behavior with TC/HDL is a crucial component of this study to highlight the risks of premature aging.

Body mass index (BMI) is a widely used metric in determining body adiposity levels and has served as an indicator for various health issues [7]. Numerous studies have shown BMI levels associated with obesity are correlated to increased risk and the likelihood of acquired cardiovascular disease and metabolic disorders throughout the aging process [7, 15]. High BMIs are associated with sedentary behavior through higher sitting times, less exercise, and altered lipid profiles [3, 16]. Despite the reported associations of BMI with aging-related issues, previous work has shown the importance of considering other metrics beyond BMI for a complete picture of one’s cardiovascular and metabolic health [17]. Our study considers this through including both BMI and TC/HDL as early health indicators for those in established adulthood.

The consensus on preventing or countering a high BMI and suboptimal TC/HDL (BMI >25 kg/m^2^ and TC/HDL >3.5 for males, >3.0 for females) is to engage in aerobic exercise each week [18, 19]. The United States Department of Health and Human Services recommends 150 minutes of moderate aerobic physical activity or 75 minutes of vigorous aerobic physical activity each week [20]. Different types of physical activity have a metabolic equivalent of task (MET) score to indicate the overall level of energy expenditure and classify the activity as low, moderate, or vigorous intensity [21]. Moderate and vigorous activities are associated with beneficial energy expenditure and can offer improvement to an individual’s health [19].

While the physical activity recommendations make it clear that any physical activity in the form of moderate or vigorous intensity is better than none, some studies have noted differences in the benefits between moderate and vigorous activity. Such studies have found that vigorous activity offers enhanced benefits in decreasing the incidence of metabolic syndrome and cardiovascular risk [19, 22, 23]. Specifically, long-term (e.g., three months or more) increases in time spent performing physical activity with healthy eating may reduce LDL and improve HDL levels [18]. Whether the negative health impacts of prolonged sitting can be significantly countered by moderate or vigorous activity is unclear. This study offers a better understanding of physical activity intensity counters on prolonged sitting as well as brings novel insight using co-twin control analysis to control confounds and understand the effects of sedentary activity and different intensities of physical activity.

In this study, we address the impacts of prolonged sitting time on cardiovascular and metabolic health indices as they relate to cardiovascular- and metabolic-related health indicators in the critical period of young adulthood that is salient to predicting later life cardiovascular and metabolic disease risk [7, 24]. Specifically, we aim to understand whether adhering to current physical activity recommendations buffers the effects of prolonged sitting on biomarkers, namely TC/HDL and BMI. Previous work has not addressed the sufficiency of current physical activity recommendations, whether it is moderate or vigorous intensity in contexts of prolonged sitting activity. This study offers a unique large sample set including twins which can be instrumental in understanding whether the effects of sitting and physical activity are better explained by familial confounds. Identical or monozygotic (MZ) twins who vary in physical activity and prolonged sitting may serve as a lens to examine activity replacement or compensation on health biomarkers, including TC/HDL which may be an early indicator or may potentiate unhealthy aging. We expect that prolonged sitting totaling most of one’s waking hours will be associated with higher TC/HDL and BMI illustrating emerging negative health impacts during early adulthood. Physical activity is expected to offer a counter to these prolonged sitting impacts, illustrated through MET minutes for moderate or vigorous activity, but not enough given the current physical activity recommendations.

## Methods

### Participants

This study included participants from the Colorado Adoption/Twin Study of Lifespan Behavioral Development and Cognitive Aging (CATSLife) recruited between July 1, 2015 and March 31, 2021 [25] (n = 1327, female = 53%, *M* age = 33.2 (SD = 4.9)). All research was approved by the Institutional Review Boards at the Institute for Behavioral Genetics–University of Colorado, Boulder, and the University of California, Riverside, respectively, and was conducted according to the APA’s ethical standards. Participants’ informed consent to participate was attained through written consent. Further, CATSLife is comprised of two separate samples. The Colorado Adoption Project (CAP) includes adopted and non-adopted siblings from adoptive and non-adoptive (“control”) families (N = 597). The Longitudinal Twin Study (LTS) is composed of individuals from same-sex monozygotic (MZ) or dizygotic (DZ) twin pairs (N = 730). Both studies recruited participants while in childhood in the state of Colorado, though 41% now live in other parts of the US. Participants in the current study were 28–49 years old at the time of blood collection. Most participants identified as non-Hispanic European American (90.1%) with a small proportion comprising Hispanic (7.2) and 2.7% representing other races/ethnicities.

To reduce the presence of confounding factors, exclusion criteria included in all analyses: current pregnancy (n = 19), more than 90 days between answering the questionnaire and completing the blood draw (n = 10), non-fasting individuals during the blood draw (n = 56), taking recognized medications that interfere with measured biomarkers [26] (n = 143, for list of contraindicating medications see S1 Table. Not participating in the blood draw and/or body measurements (n = 178 for TC/HDL and n = 162 for BMI) was also considered. The remaining participants met all inclusion criteria and were included in the analysis (n = 921 for the TC/HDL model, 937 for the BMI model).

### Measures

#### Sedentary behavior

Participants self-reported sitting behavior as part of the PhenX Toolkit protocol [27, 28]. Specifically, participants estimated, to the nearest half hour, the amount of time they spend sitting each day, including time traveling, at work, watching television, using a computer at home, and during non-television leisure time (see S1 Appendix for more information on how sitting time was calculated).

#### Physical activity

Participants were asked how much time they spent performing different types of physical activity each week, based on leisure activity screening questionnaires [29–31]. Participants were also asked to provide short, open-ended responses about the type of physical activity performed, which were assigned a MET score using double-entry coding by raters based on the 2011 Compendium of Physical Activities [21, 32]. MET scores range from 1.0 to 17.5, and scores between 3 and 6 were classified as moderate MET activities (mMETs), whereas values 6 or greater were classified as vigorous (vMETs) coinciding with previous literature [33]. These MET scores were used to calculate MET minutes per week of both moderate and vigorous physical activity. In addition, occupational physical activity was also incorporated into the calculations for moderate and vigorous MET minutes. mMETs and vMETs were both scaled by 500 MET minute increments (range for moderate MET minutes = 0–8.76, range for vigorous MET minutes = 0–11.40). The reasoning for scaling mMETs and vMETs by these increments was to better analyze the results in a meaningful and consistent way in terms of time spent performing a moderate or vigorous activity. More information regarding coding and calculations can be found in the supplementary methods (S1 Appendix) and in S1 Fig.

#### Health biomarkers

Outcomes of interest were measures of cardiovascular and metabolic health, including BMI, TC, and HDL. Most in-person testing was done prior to March 2020. BMI was calculated by taking a participant’s weight (in kilograms) divided by height squared (in meters). Weight was recorded via an analog scale and height was measured by a tape measure by a tester during an in-person visit. Fasting-state blood draws were completed during the in-person visit and a lipid panel was completed. TC was calculated by adding LDL to HDL and 20% of the TG level [9]. The TC/HDL ratio was then calculated by dividing TC by HDL (TC/HDL).

#### Covariates

Covariates included sex, age, and daily fruit and vegetable intake (FruitVegs) based on prior work supporting sex differences [34–36]. To account for nonlinear age effects on TC/HDL and BMI [37, 38], we included age squared (age^2^). Sex was dichotomized with females set as the reference. All other covariates were mean-centered. FruitVegs was indexed based on the National Cancer Institute five-factor screener from the PhenX repository [39, 40] and participants self-reported their intake frequency on several foods: (1) 100% fruit juice, (2) fruit, (3) green leafy or lettuce salad, (4) white potatoes (not including French fries), (5) cooked dried beans, (6) other vegetables, (7) tomato sauces, and (8) salsa. Responses were scaled according to daily consumption rates: never = 0; 1–3 times last month = 0.067; 1–2 times/week = 0.214; 3–4 times/week = 0.5; 5–6 times/week = 0.786; 1 time/day = 1; 2 times/day = 2; 3 times/day = 3; 4 times/day = 4; 5 or more times/day = 5.

#### Analytical strategy

The *nlme* package version 3.1–159 in *R* was used [41] to fit linear mixed-effects models (LME) for both TC/HDL and BMI. This package was used to account for possible dependency among sibling types within our sample data. Co-twin control analysis was conducted in SAS 9.4 (SAS Institute Inc., Cary, NC, USA). Tests of significance for the co-twin control analysis were confirmed via goodness of fit tests (see S2 Table).

#### Linear mixed-effects models

The initial sociodemographic base models tested for age and sex effects with TC/HDL or BMI as outcomes, adjusting for race and ethnicity:

$$Y_{ij}=B_{0_{ij}}+B_{1}(Sex)+B_{2}(Age)+B_{3}(White)+B_{4}(NonHispanic)+e_{ij},$$ [1]

$$Y_{ij}=B_{0_{ij}}+B_{1}(Sex)+B_{2}(Age)+B_{3}({Age}^{2})+B_{4}(White)+B_{4}(NonHispanic)+e_{ij},$$ [2]

The subsequent models added modifiable lifestyle factors of mMETs, vMETs, Sitting, and FruitVegs adjusting for age, sex, race and ethnicity with TC/HDL or BMI as outcomes:

$$Y_{ij}=B_{0_{ij}}+B_{1}(mMETs)+B_{2}(vMETs)+B_{3}(Sitting)+{B_{4}(FruitVegs)+B_{5}(Sex)+B}_{6}(Age)+B_{8}(White)+B_{9}(NonHispanic)+e_{ij},$$ [3]

Finally, interactions between Age, Sitting, and vMETs were tested in the final equation for TC/HDL or BMI as outcomes:

$$Y_{ij}=B_{0_{ij}}+B_{1}(mMETs)+B_{2}(vMETs)+B_{3}(Sitting)+{B_{4}(FruitVegs)+B_{5}(Sex)+B}_{6}(Age)+B_{7}(White)+B_{8}(NonHispanic)+B_{9}(Age\;x\;vMETs)+B_{10}(Age\;x\;Sitting)+B_{11}(vMETs\;x\;Sitting)+B_{12}(vMETs\;x\;Sitting\;x\;Age)+e_{ij},$$ [4]

For each equation, (*Y*_*ij*_) is the respective outcome (i.e. TC/HDL or BMI), *i* is the ith individual in jth family, and $B_{0_{ij}}$and *e*_*ij*_ reflect the intercept and the residual, respectively. The LME models included race (White) and ethnicity (NonHispanic) adjustments. The LME models further adjusted for differential sibling dependencies by estimating between- and within-family random effects for each of the four sibling/family types (adoptive, control, DZ, MZ) [42]. Finally, a Cohen’s d effect size of the LME was calculated for both the TC/HDL and BMI by multiplying the unstandardized beta of vigorous MET minutes by two (representing 1000 vigorous MET minutes, a value close to the vigorous MET minutes standard deviation) and then dividing by the outcome standard deviation, resulting in an approximation of a standardized regression coefficients.

#### Co-twin control analysis

Co-twin control analyses were conducted using twin pairs from the LTS subsample to evaluate confounding genetic/familial versus environmental impacts [43]. Using available complete pairs (MZ twin pairs n = 98, DZ twin pairs n = 91), a co-twin control analysis was completed using maximum likelihood estimation. Both TC/HDL and BMI were tested as outcomes. The twin pair mean was calculated for Sitting, mMETs, vMETs, FruitVegs representing the familial risk. Each twin difference was calculated by taking the twin’s unique value subtracted from the respective twin pair mean to indicate the risk difference after accounting for familial risk. Fixed effects included the twin pair mean for Sitting, mMETs, vMETs, or FruitVegs, the respective twin difference from the twin pair mean, and the zygosity interactions (DZs coded as 0, MZs coded as 1) with both the pair mean and difference. Models were also adjusted for age and sex. Expected patterns of findings consistent with environmental/exposure association are as significant differences noted in the within effects of the analysis with consistent results across zygosity where little to no difference exists between MZs and DZs. Expected patterns of findings consistent with genetic or familial confounding include significances of between effects or significant findings by zygosity interactions where MZ twins pairs show significant or strong trends in an outcome compared to DZ twins.

#### Discordant monozygotic twin pairs

An available extension of the co-twin control design is a sensitivity analysis on the discordant MZ twin pairs. By examining MZ pairs where siblings differed on activity engagement, we can elucidate whether exercise mitigates the effects of prolonged sitting time. We conducted a subtest of MZ pairs with a small sample (N = 40 MZ pairs) that differed (i.e. discordant) on vigorous exercise and sitting time. The difference in sitting hours per week, hours per week of vigorous physical activity, and the TC/HDL were calculated between pairs. Blood draws for each MZ twin in the sample were completed during the same age as their co-twin (Mean difference = 22.4 days, median = 0 days). For MZ pairs to be considered discordant there had to exist a difference of at least 3.5 hours of sitting per week (i.e. 30 minutes per day) and a difference of over 15 minutes of vigorous exercise per week. Based on the sitting and vigorous exercise times, MZ pairs were placed into two groups. One group included pairs where the twin that sat less and completed more vigorous exercise compared to their co-twin were placed into this group, known as the “Active Replacer” group. The Active Replacer group (n = 20) represents a MZ pair where one twin essentially replaces the unhealthy behavior of sitting with some amount of vigorous exercise. The other group, known as the “Active Compensator” group (n = 20), included pairs where the twin that sat more also completed more vigorous exercise compared to their co-twin. This group represents a MZ pair where one twin is compensating high levels of sitting time with vigorous exercise compared to their co-twin, potentially offering some level of counter on their overall health to their higher levels of sitting. The tradeoff between sitting time and vigorous exercise was calculated for either group by taking the pair difference in hours per week of vigorous exercise divided by the difference in hours per week of sitting. For example, if a twin in the Active Replacer group sat 20 hours less a week and completed 2 hours more vigorous exercise a week, the tradeoff value would be calculated as 0.1 (2 / 20). These values were then multiplied by 60 to rescale from hours to minutes. This would mean for the example case that for each hour less of sitting per week, the twin completed 6 minutes of vigorous exercise in its place (0.1 * 60 = 6). For the Active Compensator group, this example would translate to each additional hour of sitting, the twin completes 6 minutes of vigorous exercise. To minimize outliers, MZ pairs with vigorous physical activity ≥2.5 minutes of vigorous activity for each hour of sitting were considered and values were winsorized to 60 minutes.

## Results

### Descriptive statistics

Descriptive statistics for key measures are reported in Table 1. On average, participants reported sitting 60.07 hours per week, or 8.58 hours per day. Sitting hours were approximately normally distributed with no mean-level sex differences. vMETs, FruitVegs, BMI, and TC/HDL were significantly higher in males compared to females (*p* *≤* .005). No other sex differences were found. Within the sample, the correlation of BMI and TC/HDL values was found to be moderate with a Spearman’s rank correlation coefficient of 0.41.

**Table 1:** Descriptive statistics for primary variables and covariates.

	Male			Female			All			Sex
	*M*	*SD*	*N*	*M*	*SD*	*N*	*M*	*SD*	*N*	*p*-value
**mMETs**	778.4	806.2	553	719.9	706.5	546	479.3	758.5	1099	.289
**vMETs**	903.8	1163.9	553	708.7	1031.7	546	806.9	1104.0	1099	.004*
**Sitting**	61.1	21.0	543	59.0	22.2	531	60.1	21.6	1074	.091
**FruitVegs**	3.2	2.2	545	6.0	4.0	534	4.6	3.5	1079	< .001*
**TC/HDL**	3.6	1.0	479	3.0	0.8	460	3.3	0.9	939	< .001*
**BMI**	27.5	5.5	515	26.4	6.5	501	26.9	6.1	1016	.005*

**Notes:** mMETs = Unscaled Moderate MET Minutes per week, vMETs = Unscaled Vigorous MET Minutes per Week, Sitting = Hours per Week, FruitVegs = Fruit and Vegetable Intake in Cups, TC/HDL = Total Cholesterol to HDL Ratio, BMI = Body Mass Index, sex difference *p*-values were calculated using an unpaired t-test.

*p < 0.05.

### Linear mixed-effect (LME) models

**Fig 1:**
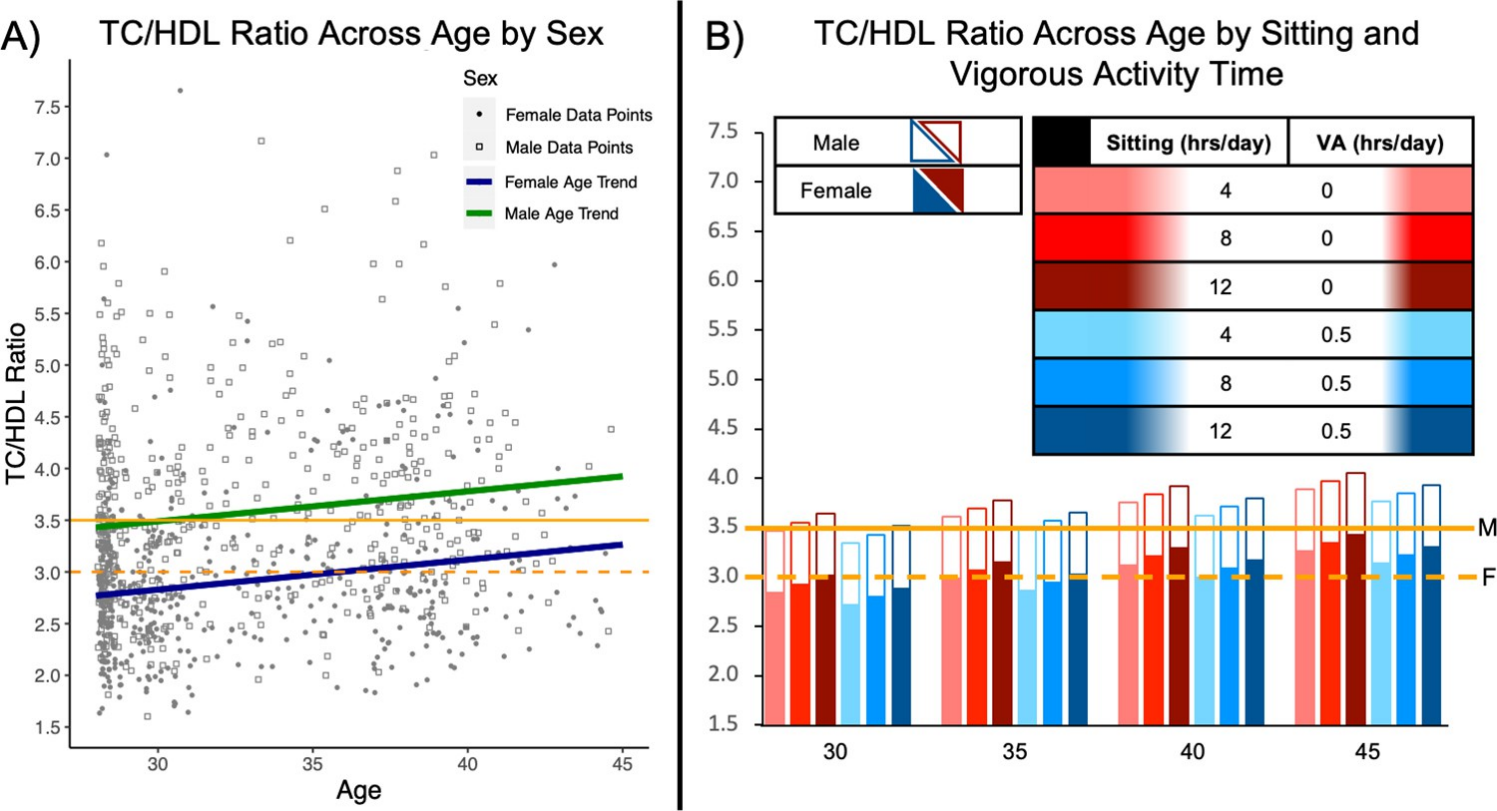
Linear mixed effect model results for TC/HDL ratio by age, sex, and modifiable behaviors. (A) Visual representation of linear mixed effects model results for TC/HDL Ratio by age and sex (n = 921). TC/HDL Ratio <3.5 for males and <3.0 for females is classified as optimal level. Moderate TC/HDL Ratio levels are between 3.5–5.0 for males indicated by a solid yellow line and 3.0–4.4 for females indicated by a dashed yellow line. High TC/HDL ratio values are >5.0 for males and >4.4 for females. Trend lines by age are also shown across early and mid-adulthood with the blue line representing females and the green line representing males. (B) Results of linear mixed effects model for TC/HDL Ratio including modifiable risk factors of sitting time and vigorous physical activity (VA) (n = 921). Moderate TC/HDL for males is indicated by the solid yellow line. Moderate TC/HDL for females is indicated by the dashed yellow line.

**Fig 2:**
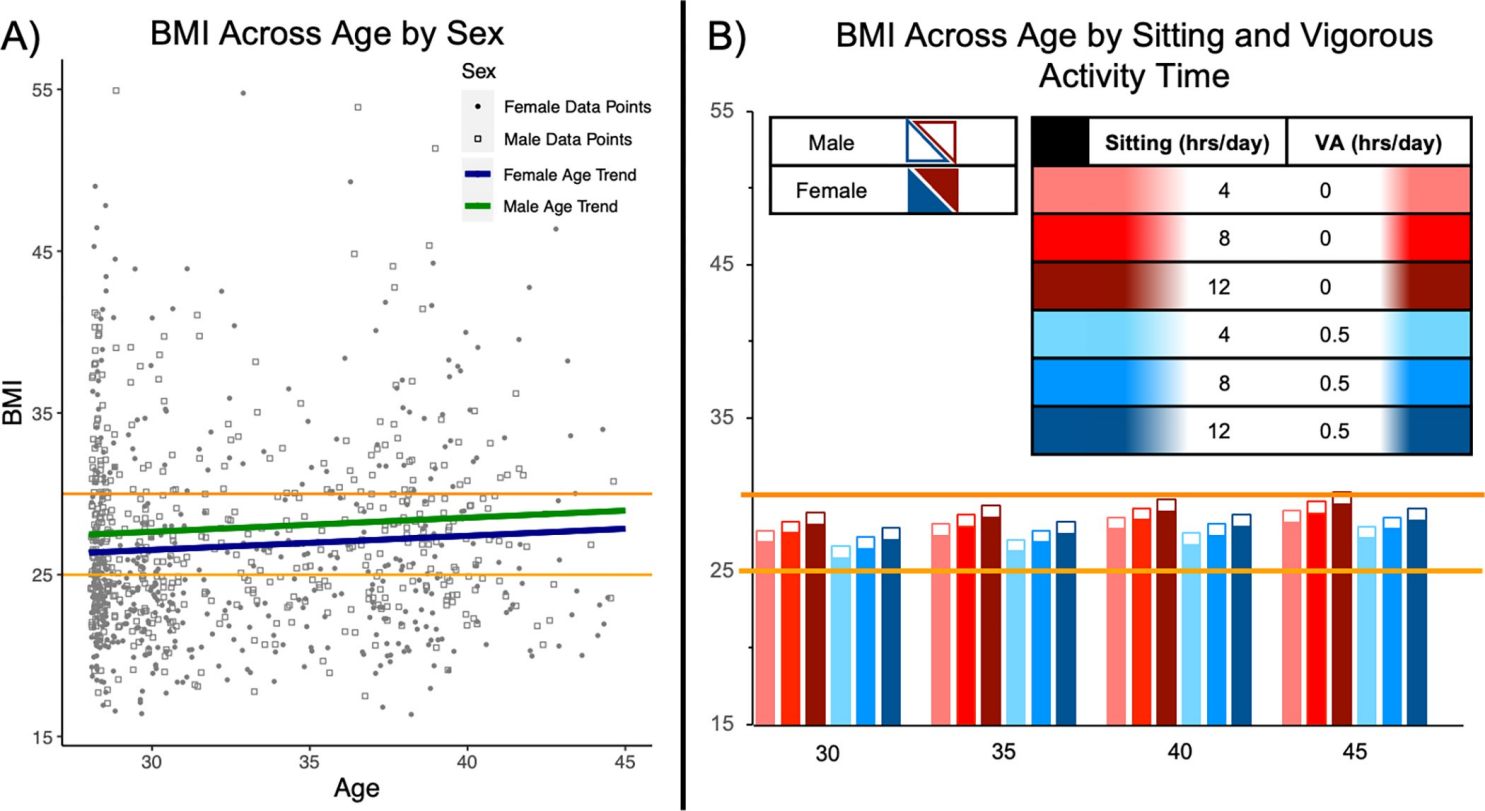
Linear mixed effect model results for BMI by age, sex, and modifiable behaviors. (A) Visual representation of linear mixed effects model results for BMI by age and sex (n = 937). BMI levels for normal BMI are between 18.5–24.9 kg/m^2^. Overweight BMI is between 25–29.9 kg/m^2^ and is illustrated by the solid yellow line. Obese BMI is between 30–34.9 kg/m^2^ and is indicated by the solid red line. Trend lines by age are also with the blue line representing females and the green line representing males. (B) Results of linear mixed effects model for BMI including modifiable risk factors of sitting time and vigorous physical activity (VA) (n = 937). Overweight BMI is indicated by the solid yellow line and obese BMI is indicated by the solid orange line.

Results from the sociodemographic base models for both TC/HDL and BMI outcomes included significant age (B = 0.029, *p* = < .001 for TC/HDL and B = 0.087, *p* = .004 for BMI) and sex effects (B = 0.660, *p* = < .001 for TC/HDL and B = 1.121, *p* = .006) (S3 Table). Race and ethnicity were non-significant but retained in subsequent models (*p*≥ 0.232). Entering age-squared effects showed potential non-linear trends but were not significant and were not retained in subsequent models (B = -0.002, *p* = .211 for TC/HDL and B = 0.008, *p* = .383 for BMI; see S3 Table). The unstandardized regression parameters for the model that are shown in Table 2. TC/HDL was associated with age, sex, vMETs, and sitting hours per week. Model results indicate higher TC/HDL and BMI with older ages across established adulthood as indicated in Figs 1A and 2A. The age effects for TC/HDL and BMI showed little attenuation (-0.001 for both outcomes) after adding in the modifiable risk factors of sitting, physical activity, and healthy diet. Results suggested individuals with greater vMETs had a lower TC/HDL ratio. In contrast, being older, male, and reporting longer sitting times were associated with worse TC/HDL. Although healthy eating (FruitVegs) and greater mMETs were associated with a lower TC/HDL ratio, the effects were nonsignificant. TC/HDL model results are illustrated in Fig 1B where estimates show age trends of higher TC/HDL values with older ages. Notably, within males and within females, patterns suggested a 30-year-old individual sitting for 4 hours on average a day would have comparable TC/HDL to that of a 35-year-old individual sitting the same amount per day but performing at least 30 minutes of vigorous exercise each day. Alternatively stated, in terms of the age-equivalent benefit of 30-minutes vigorous exercise at equivalent sitting times of 4 hours: those performing 30 minutes of vigorous exercise daily at age 35 years had lower TC/HDL values equivalent to sedentary individuals 5 years younger.

**Table 2:** Linear mixed-effects parameters for TC/HDL and BMI.

TC_HDL ~ mMETs + vMETs + Sitting + FruitVegs + Age + Sex + Ethnicity1 + Ethnicity2					BMI ~ mMETs + vMETs + Sitting + FruitVegs + Age + Sex + Ethnicity1 + Ethnicity2				
	*B*	*SE*	DF	p-value		*B*	*SE*	DF	*p*-value
Intercept	3.025	0.125	600	< .001*	Intercept	27.822	0.857	608	< .001*
mMETs	-0.026	0.018	312	.163	mMETs	-0.206	0.121	320	.090
vMETs	-0.037	0.012	312	.003*	vMETs	-0.306	0.084	320	< .001*
Sitting	0.003	0.001	312	.021*	Sitting	0.021	0.009	320	.013*
FruitVegs	-0.015	0.009	312	.093	FruitVegs	-0.133	0.059	320	.026*
Age	0.028	0.006	312	< .001*	Age	0.086	0.042	320	.043*
Male	0.628	0.064	312	< .001*	Male	0.770	0.437	320	.079
White	-0.043	0.138	312	.755	White	-0.842	0.931	320	.366
Non-Hispanic	0.075	0.147	312	.608	Non-Hispanic	0.245	1.002	320	.807
Individual *N*	921				Individual *N*	937			
Families *N*	601				Families *N*	609			

*Notes*: Equations are listed along with values for each main effect predictor model. DF = degrees of freedom, mMETs = Moderate MET minutes per week, vMETs = Vigorous MET minutes per week, Sitting = Hours per Week, FruitVegs = Fruit and Vegetable Intake in Cups.

*p < 0.05.

Similar patterns were seen for BMI with healthy eating and physical activity showing negative relationships with BMI, but significant associations were different from the TC/HDL model. In addition to vMETs, FruitVegs was also a significant predictor of BMI. Other predictors for BMI all had positive associations with BMI in the model, with Sitting as the only positive association that was also statistically significant. BMI model results are illustrated in Fig 2B where estimates show age trends of higher BMI values at older ages. Notably, within males and within females, patterns suggested that a 30-year-old individual sitting 4 hours on average a day would have a comparable BMI to that of a 40-year-old individual sitting the same amount per day but performing at least 30 minutes of vigorous activity (Fig 2B). Alternatively stated, in terms of the age-equivalent benefit of 30-minutes vigorous exercise at equivalent sitting times of 4 hours: those performing 30 minutes of vigorous exercise daily at age 30 years had lower BMI values equivalent to sedentary individuals 10 years younger. Our results indicate an individual sitting on average 8.5 hours per day performing at or below the current exercise recommendations could enter a moderate TC/HDL risk category in early adulthood.

S4 Table shows the results of the model with age, sitting, and vMETs interactions where none of the interactions were found to have statistical significance. Vigorous activity was a more effective counter in both tested biomarkers with an effect size of -0.08 for TC/HDL (for 1000 vigorous MET minutes per week more, which is equivalent to jogging 20 minutes a day) and -0.11 for BMI (for 1000 vigorous MET minutes per week more).

### Co-twin control analysis

For TC/HDL, only the within-pair effect was significant for sitting and vigorous physical activity (see Table 3). The within-pair effect did not significantly differ by zygosity, consistent with an exposure effect. However, the MZ within-pair effect for vigorous activity was smaller relative to the general within-pair effect suggesting partial confounding in TC/HDL. Although the between-pair effects were not significant; the effects were in the same direction. Parameters are reported in Table 3 for vigorous exercise and BMI. We only found significant between-pair effects for vigorous exercise which did not differ by zygosity. The within-pair effects while in the same direction were attenuated and non-significant for both DZ and MZ pairs, consistent with familial confounding rather than an exposure effect.

**Table 3:** Co-twin control analysis–between-within results.

TC/HDL & Sitting	B	SE	t Value	Pr > |t|	F Value	Pr > F
Intercept	3.101	0.077	40.28	<0.001	.	.
Sitting Between Pair Effect	0.004	0.005	0.77	0.443	0.47	0.492
Sitting Between Pair Effect · MZ	-0.004	0.007	-0.53	0.594	0.28	0.594
Sitting Within Pair Effect	0.011	0.004	2.91	0.004*	7.48	0.007*
Sitting Within Pair Effect · MZ	-0.009	0.005	-1.86	0.064	3.47	0.064
Age	0.002	0.042	0.04	0.965	0	0.965
Sex	0.577	0.103	5.59	<0.001*	31.25	<0.001*
TC/HDL & vMETs	B	SE	t Value	Pr > |t|	F Value	Pr > F
Intercept	3.146	0.093	33.82	<0.001	.	.
Vigorous PA Between Pair Effect	-0.015	0.033	-0.47	0.639	0.56	0.455
Vigorous PA Between Pair Effect · MZ	-0.009	0.038	-0.23	0.820	0.05	0.820
Vigorous PA Within Pair Effect	-0.048	0.043	-1.14	0.257	5.09	0.025*
Vigorous PA Within Pair Effect · MZ	-0.016	0.050	-0.32	0.753	0.1	0.753
Age	0.007	0.042	0.16	0.871	0.03	0.871
Sex	0.580	0.103	5.62	<0.001*	31.56	<0.001*
BMI & vMETs	B	SE	t Value	Pr > |t|	F Value	Pr > F
Intercept	26.803	0.629	42.64	<0.001	.	.
Vigorous PA Between Pair Effect	-0.457	0.204	-2.24	0.026*	7.41	0.007*
Vigorous PA Between Pair Effect · MZ	-0.064	0.261	-0.25	0.806	0.06	0.806
Vigorous PA Within Pair Effect	-0.082	0.268	-0.3	0.761	0.26	0.609
Vigorous PA Within Pair Effect · MZ	-0.001	0.321	0	0.998	0	0.998
Age	-0.357	0.280	-1.27	0.205	1.62	0.205
Sex	1.801	0.701	2.57	0.011*	6.59	0.011*

*Notes*: n = 189 twin pairs for all models. Chi-squared goodness of fit tests were considered when t and F values diverged (see S2 Table).

*p < 0.05.

### Discordant MZ twin pairs

**Fig 3:**
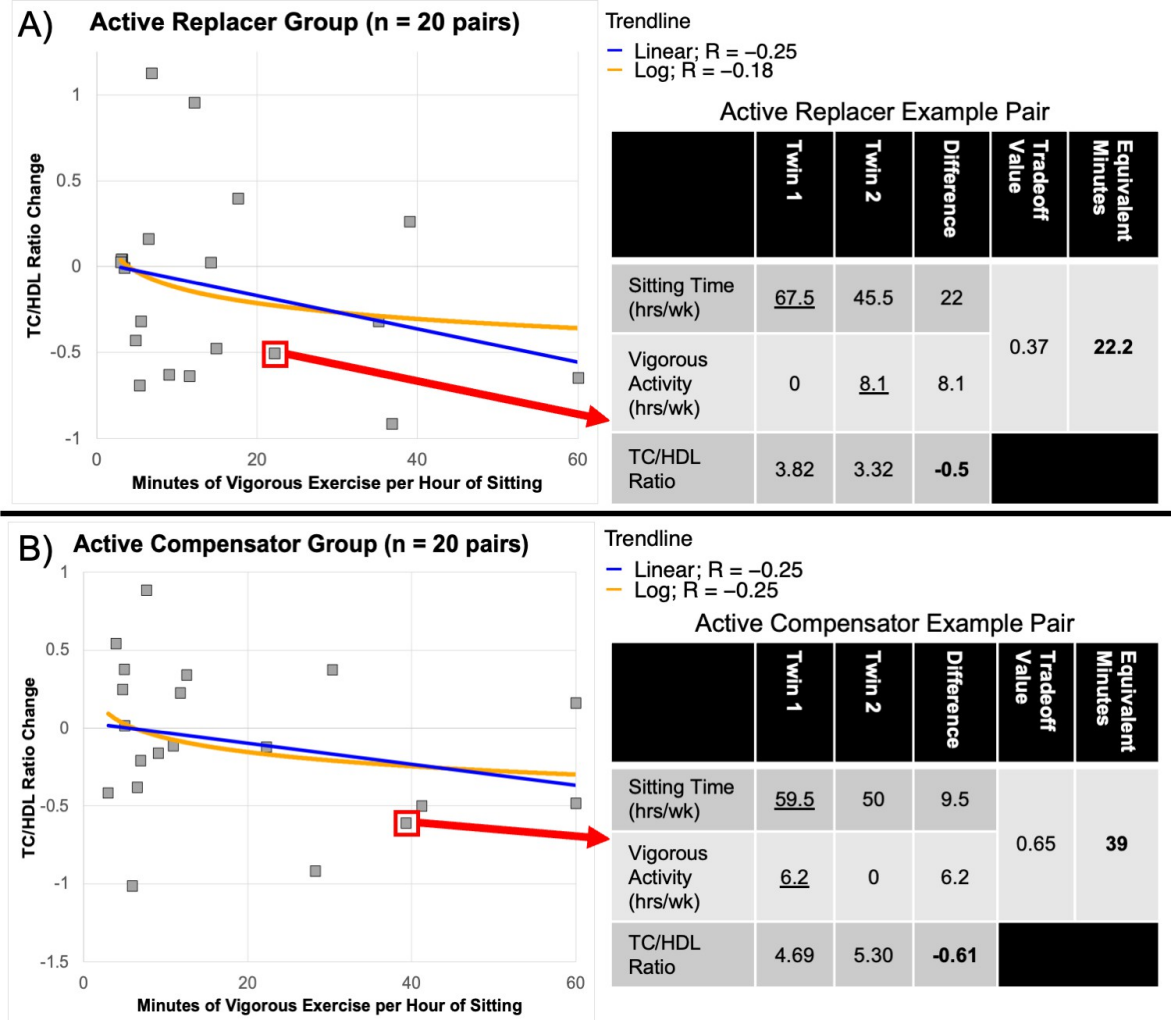
TC/HDL change within discordant monozygotic twin pairs. A) Change in TC/HDL Ratio observed between monozygotic twin pairs where the twin with a lower sitting time also completes a higher level of vigorous physical activity (n = 20 twin pairs). The x-axis represents minutes of vigorous physical activity that replace each hour less of sitting between the twin pairs. B) Change in TC/HDL Ratio observed between monozygotic twin pairs where the twin with a higher sitting time also completes a higher level of vigorous physical activity (n = 20 twin pairs). The x-axis represents additional minutes spent completing vigorous physical activity for each additional hour of sitting between the twin pairs.

Trendlines for MZ pair groups are shown in Fig 3. Trendlines for both discordant groups share a similar downward trend, however, the Active Replacer group has a noticeably lower starting point close to a 0.0 TC/HDL change compared to the Active Compensator group which started with a positive TC/HDL change. For the Active Replacer group trend, the twin sitting less and engaging in more vigorous physical activity had lower TC/HDL, particularly when at least 10 minutes of vigorous activity were replaced for each hour of sitting. For the Active Compensator group similar trends were noted; however, to reach similar TC/HDL levels as the substitution group, individuals would need to engage in well over 10 minutes of vigorous activity.

## Discussion

We examined whether physical activity buffers high levels of sitting time on cardiovascular and metabolic health indices of TC/HDL and BMI in a sample of young adults. The expected negative associations of sitting on health indices were apparent in this relatively healthy period of adulthood. However, meeting the current physical activity recommendations did not buffer the impacts of sitting on BMI or TC/HDL fully, although engagement in vigorous physical activity is associated with lower, healthier levels. For females in their early 30s and males in their late 20s, the TC/HDL is shown to cross into the moderate cardiac risk territory when sitting 8.5 hours per day even after meeting current physical activity recommendations. Performing vigorous physical activity reveals a notable age-equivalent benefit with any level of sitting where an individual exercising 30 minutes of day of vigorous intensity exercise has comparable TC/HDL and BMI values to someone 5 or 10 years younger respectively sitting the same amount of time without any vigorous physical activity. Our findings suggest maintaining sedentary behavior throughout young adulthood may contribute to later cardiovascular and metabolic disease susceptibility [7, 15]. The co-twin control analysis sensitivity analyses with the MZ pairs further illustrates the importance of additional vigorous physical activity in place of sitting time or in addition to prolonged sitting time to improve one’s TC/HDL. Failing to disrupt sedentary behavior could set a course towards poorer health and functioning across the lifespan, particularly since once disease onset occurs for many chronic conditions, disease maintenance will be the primary focus of health intervention instead of recovery. This has been illustrated in previous work implicating elevated BMI and dyslipidemia measurements in early adulthood linked to adverse impacts occurring later in life as they relate to issues such as coronary heart disease, stroke, and other major health issues [44–46]. Given these links, early intervention of suboptimal BMI and TC/HDL values is critical to prevent a multitude of health-related issues past early adulthood.

While TC/HDL and BMI were found to have a positive correlation (Spearman’s rank correlation coefficient = 0.41 in our sample indicate a moderate correlation), it is important to consider both within the analysis due to over 33% of the overall sample exhibiting either a healthy BMI and unhealthy TC/HDL or an unhealthy BMI and healthy TC/HDL. By including early-risk biomarkers highly relevant to metabolic (BMI) and cardiovascular (TC/HDL) health, a more comprehensive view of behavioral associations can be attained. The TC/HDL and BMI showed positive associations with greater sitting, consistent with the literature [16]. Specifically, vigorous activity and sitting time were significantly associated with TC/HDL and BMI, after accounting for sociodemographic and dietary covariates. Vigorous activity partially countered increased TC/HDL and BMI levels but could not fully buffer the negative impacts of prolonged sitting. While moderate activity showed similar patterns, it was not significant. The benefit of engaging in vigorous over moderate activity is in line with previous studies [19, 22, 23], however, our findings are among the first to show this for sitting behavior as well as offer a clear picture of the amount of physical activity as they relate to the current exercise recommendations. More sitting time was significantly associated with an increase in TC/HDL and BMI. Our observations are supportive of the negative impacts of prolonged sitting totaling over half of one’s waking hours given the significant associations with major risk factors for unhealthy aging. Males are more apt to have a higher cardiovascular risk compared to females, particularly in young adulthood [47] and our findings showed significant sex effects as well as age effects for the TC/HDL. Our results uniquely show that the early warning signs accompanying prolonged sitting can put one in elevated risk categories for cardiovascular and metabolic issues even at young, typically healthy ages. Our results suggest that males in their early 30’s would need to surpass the current exercise recommendations, particularly if sitting for more than 8 hours per day in order to avoid falling into the moderate risk category for TC/HDL. Our results also suggest females in their mid 30’s would also need to surpass current exercise recommendations to remain in the optimal TC/HDL range. BMI model results for both males and females show that across early adulthood along with a healthy diet intake, exercise past the current guidelines would be beneficial to maintaining optimal metabolic health. Entering the moderate TC/HDL risk range as well as having a BMI outside of normal range throughout established adulthood has been shown across previous work to be associated with aging-related cardiac and metabolic events and illnesses [48–50]. This emphasizes previous work on the importance of understanding how behaviors and measures in early adulthood can impact aging and health later on in life [51]. Sitting less throughout the day, getting much more vigorous exercise, or a combination of both may be necessary to reduce the risk of premature aging in early adulthood.

Our sample allows for a unique analysis to examine the differences between siblings in a twin pair on health. The co-twin control approach is considered a quasi-experimental design that offers unique insights into behavioral or environmental exposures while controlling for shared genetic and common environment between siblings [25]. Less sitting and more vigorous physical activity were associated with better TC/HDL. This is in line with prior work [19], however, most studies rely on correlational evidence or cannot control for selection factors in physical activity engagement [19, 22, 23]. Our analysis revealed causal evidence of vigorous activity impact on TC/HDL, with partial confounding for sitting due to differences noted between MZ and DZ twins. We did not find causal evidence between vigorous activity and BMI. Rather, between-pair and within-pair patterns suggest genetic liability and shared environmental factors are responsible for these associations which is similar to other related work [52, 53]. Our results call for further work, particularly in understanding the exposure effects in one’s unique environment on the TC/HDL.

As an extension of the co-twin control analysis, we conducted a discordant MZ twin subtest to see if physical activity can ameliorate the deleterious effects of prolonged sitting. While our study did not allow for intervention as a case-control study would, the MZ twins is a powerful exploratory method and can offer insights most studies cannot test. Vigorous activity of more than 10 minutes for each additional hour of sitting per week or in place of each hour of sitting improves the cardiovascular biomarker TC/HDL, with our results predicting that individuals sitting upwards of 8 hours per day, over 80 minutes of vigorous activity would be needed each week to stay within an optimal TC/HDL range. Hence increasing vigorous activity or reducing sitting times may be factors to consider in policy guidelines, with emphasis given to young adults to boost vigorous activity for those who do not have the ability to reduce the time spent sitting throughout the day. Further work is warranted to link both the vigorous activity replacement as well as compensating vigorous activity with lowering these and other early aging biomarkers.

Our study primarily focused on physical engagement or the lack thereof, on health yet another important consideration was diet. Fruit and vegetable consumption was significant for BMI, but not for TC/HDL. Both health indicators are well-documented to be negatively correlated with fruit and vegetable consumption [18]. While fruit and vegetable intake is a known predictor of healthy weight and BMI, why we did not find a significant link with TC/HDL is unclear. Our consumption measure may not be an ideal method to capture diet. Consumption being isolated to those specific food groups, while not weighing intake of meat and dairy animal products could contribute to our patterns. Our work does not critique current diet recommendations in the same vein as physical activity. More comprehensive diet measures would be required to properly evaluate if diet can offset consequences from prolonged sitting or the extent consumption may differ due to sitting behavior. Healthy eating remains an important lifestyle factor in conjunction with exercise, as decades of health science have shown [18], how diet may interact and influence sitting behaviors will require future investigation.

While our study showed significant impacts of prolonged sitting during young adulthood on key health indicators, limitations should be noted. Our data was calculated using self-reported data from CATSLife participants which may not be consistent with actual time spent doing such behaviors as sitting, physical activity, or dietary intake. Future studies using accelerometer or fitness tracker devices can elaborate on these findings to further elaborate the accuracy of self-reported data. Our sample included young to middle-aged adults with no selection on activity engagement, and 52.8% reported performing vigorous activity. We cannot give any inference to individuals who may have been medically advised against performing vigorous or certain types of physical activity. Also, we cannot make inferences based on these results for persons using a mobility aid or device. Finally, while our sensitivity analyses of race and ethnicity were non-significant, our sample has limited diversity to make conclusions, but important differences may still exist in sitting time, health status, and exercise interventions.

Our study shows the importance of incorporating vigorous physical activity into our daily routines to minimize or offset the health consequences of prolonged sitting. Particularly, adopting these health regimes by the end of early adulthood to prevent reaching cardiovascular and metabolic disease risk levels that promote unhealthy aging. Future work further detailing the contribution of genetic and environmental effects on the role of sedentariness is warranted. With increased sitting time illustrating sedentary behavior being a new norm across much of society, the focus must be shifted to ways of countering these long-term negative impacts. Sitting is unavoidable for many people, however, health consequences from such behavior are not inescapable. Exercise can counteract these effects and incorporating more vigorous activity would be needed as sitting time increases. Our results challenge recommended physical activity and we suggest guidelines need to be adjusted to account for sitting time throughout the day. Sitting less, healthier eating, and focusing on increasing the amount of vigorous activity need to be clear messaging points to the public to provide a succinct and impactful change to sedentary habits.

## Supporting information

S1 Appendix Calculation and adjustments for modifiable behaviors of sitting and exercise. Methodology and process of calculating sitting time and exercise based on CATSLife participant self-report responses.
(PDF)

S1 Table Contraindicating medications used for exclusion criteria. Table of medication families and specific medications that, if a participant reported taking, they were excluded from the biomarker analysis due to contraindications supported by the literature.
(PDF)

S2 Table Fit statistics for co-twin control analysis. Co-twin control change in -2 log likelihood fit statistics based on removal of specific effects or interactions.
(PDF)

S3 Table LME parameters for TC/HDL and BMI–age, age^2^ and sex. Results of LME models including the variable of Age^2^. Results indicate no significances for Age^2^.
(PDF)

S4 Table LME parameters for TC/HDL and BMI–sitting and vigorous physical activity interactions. Results of LME models including behavioral interactions and interactions with age. Results indicate no significances for these interactions.
(PDF)

S1 Fig MET minutes adjustment process. Visual representation of the adjustment process for calculating moderate and vigorous MET minutes per week for participants.
(PDF)

## References

[1] Meyer J, Herring M, McDowell C, Lansing J, Brower C, Schuch F, et al. Joint prevalence of physical activity and sitting time during COVID-19 among US adults in April 2020. Prev Med Rep. 2020;20:101256. Epub 20201127. doi: 10.1016/j.pmedr.2020.101256 ; PubMed Central PMCID: PMC7695441. 33282638 PMC7695441

[2] Hwang CL, Chen SH, Chou CH, Grigoriadis G, Liao TC, Fancher IS, et al. The physiological benefits of sitting less and moving more: Opportunities for future research. Prog Cardiovasc Dis. 2022;73:61–6. Epub 20210113. doi: 10.1016/j.pcad.2020.12.010 ; PubMed Central PMCID: PMC8628304. 33453285 PMC8628304

[3] Stamatakis E, Gale J, Bauman A, Ekelund U, Hamer M, Ding D. Sitting Time, Physical Activity, and Risk of Mortality in Adults. J Am Coll Cardiol. 2019;73(16):2062–72. doi: 10.1016/j.jacc.2019.02.031 . 31023430

[4] Mat Azmi ISM, Wallis GA, White MJ, Puig-Ribera A, Eves FF. Desk based prompts to replace workplace sitting with stair climbing; a pilot study of acceptability, effects on behaviour and disease risk factors. BMC Public Health. 2022;22(1):1985. Epub 20221031. doi: 10.1186/s12889-022-14393-1 . 36316656 PMC9620615

[5] Ekelund U, Steene-Johannessen J, Brown WJ, Fagerland MW, Owen N, Powell KE, et al. Does physical activity attenuate, or even eliminate, the detrimental association of sitting time with mortality? A harmonised meta-analysis of data from more than 1 million men and women. Lancet. 2016;388(10051):1302–10. Epub 20160728. doi: 10.1016/S0140-6736(16)30370-1 . 27475271

[6] Vallance JK, Gardiner PA, Lynch BM, D’Silva A, Boyle T, Taylor LM, et al. Evaluating the Evidence on Sitting, Smoking, and Health: Is Sitting Really the New Smoking? Am J Public Health. 2018;108(11):1478–82. Epub 20180925. doi: 10.2105/AJPH.2018.304649 ; PubMed Central PMCID: PMC6187798. 30252516 PMC6187798

[7] Meigs JB, Wilson PW, Fox CS, Vasan RS, Nathan DM, Sullivan LM, et al. Body mass index, metabolic syndrome, and risk of type 2 diabetes or cardiovascular disease. J Clin Endocrinol Metab. 2006;91(8):2906–12. Epub 20060530. doi: 10.1210/jc.2006-0594 . 16735483

[8] Xu J, Murphy S, Kochanek K, Arias E. Deaths: Final Data for 2019. National Vital Statistics Reports: Division of Vital Statistics, 2021 Contract No.: 8.

[9] Kinosian B, Glick H, Garland G. Cholesterol and coronary heart disease: predicting risks by levels and ratios. Ann Intern Med. 1994;121(9):641–7. doi: 10.7326/0003-4819-121-9-199411010-00002 . 7944071

[10] Cooney MT, Dudina A, De Bacquer D, Wilhelmsen L, Sans S, Menotti A, et al. HDL cholesterol protects against cardiovascular disease in both genders, at all ages and at all levels of risk. Atherosclerosis. 2009;206(2):611–6. Epub 20090319. doi: 10.1016/j.atherosclerosis.2009.02.041 . 19375079

[11] Calling S, Johansson SE, Wolff M, Sundquist J, Sundquist K. Total cholesterol/HDL-C ratio versus non-HDL-C as predictors for ischemic heart disease: a 17-year follow-up study of women in southern Sweden. BMC Cardiovasc Disord. 2021;21(1):163. Epub 20210405. doi: 10.1186/s12872-021-01971-1 ; PubMed Central PMCID: PMC8020530. 33820540 PMC8020530

[12] Millan J, Pinto X, Munoz A, Zuniga M, Rubies-Prat J, Pallardo LF, et al. Lipoprotein ratios: Physiological significance and clinical usefulness in cardiovascular prevention. Vasc Health Risk Manag. 2009;5:757–65. Epub 20090918. ; PubMed Central PMCID: PMC2747394. 19774217 PMC2747394

[13] Wannamethee SG, Shaper AG, Ebrahim S. HDL-Cholesterol, total cholesterol, and the risk of stroke in middle-aged British men. Stroke. 2000;31(8):1882–8. doi: 10.1161/01.str.31.8.1882 . 10926951

[14] Takahashi O, Glasziou PP, Perera R, Shimbo T, Suwa J, Hiramatsu S, et al. Lipid re-screening: what is the best measure and interval? Heart. 2010;96(6):448–52. Epub 20090614. doi: 10.1136/hrt.2009.172619 . 19528038

[15] Lee J. Influences of Cardiovascular Fitness and Body Fatness on the Risk of Metabolic Syndrome: A Systematic Review and Meta-Analysis. Am J Health Promot. 2020;34(7):796–805. Epub 20200520. doi: 10.1177/0890117120925347 . 32431155

[16] Pinto Pereira SM, Ki M, Power C. Sedentary behaviour and biomarkers for cardiovascular disease and diabetes in mid-life: the role of television-viewing and sitting at work. PLoS One. 2012;7(2):e31132. Epub 20120209. doi: 10.1371/journal.pone.0031132 ; PubMed Central PMCID: PMC3276501. 22347441 PMC3276501

[17] Wang JS, Xia PF, Ma MN, Li Y, Geng TT, Zhang YB, et al. Trends in the Prevalence of Metabolically Healthy Obesity Among US Adults, 1999–2018. JAMA Netw Open. 2023;6(3):e232145. Epub 20230301. doi: 10.1001/jamanetworkopen.2023.2145 ; PubMed Central PMCID: PMC9999245. 36892842 PMC9999245

[18] Katcher HI, Hill AM, Lanford JL, Yoo JS, Kris-Etherton PM. Lifestyle approaches and dietary strategies to lower LDL-cholesterol and triglycerides and raise HDL-cholesterol. Endocrinol Metab Clin North Am. 2009;38(1):45–78. doi: 10.1016/j.ecl.2008.11.010 . 19217512

[19] Eijsvogels TMH, Maessen MFH. Exercise for Coronary Heart Disease Patients: Little Is Good, More Is Better, Vigorous Is Best. J Am Coll Cardiol. 2017;70(14):1701–3. doi: 10.1016/j.jacc.2017.08.016 . 28958325

[20] Tucker JM, Welk GJ, Beyler NK. Physical activity in U.S.: adults compliance with the Physical Activity Guidelines for Americans. Am J Prev Med. 2011;40(4):454–61. doi: 10.1016/j.amepre.2010.12.016 . 21406280

[21] Craig CL, Marshall AL, Sjöström M, Bauman AE, Booth ML, Ainsworth BE, et al. International physical activity questionnaire: 12-country reliability and validity. Med Sci Sports Exerc. 2003;35(8):1381–95. doi: 10.1249/01.MSS.0000078924.61453.FB . 12900694

[22] Swain DP. Moderate or vigorous intensity exercise: which is better for improving aerobic fitness? Prev Cardiol. 2005;8(1):55–8. doi: 10.1111/j.1520-037x.2005.02791.x . 15722695

[23] López-Martínez S, Sánchez-López M, Solera-Martinez M, Arias-Palencia N, Fuentes-Chacón RM, Martínez-Vizcaíno V. Physical activity, fitness, and metabolic syndrome in young adults. Int J Sport Nutr Exerc Metab. 2013;23(4):312–21. Epub 20121207. doi: 10.1123/ijsnem.23.4.312 . 23239681

[24] Zhang Y, Vittinghoff E, Pletcher MJ, Allen NB, Zeki Al Hazzouri A, Yaffe K, et al. Associations of Blood Pressure and Cholesterol Levels During Young Adulthood With Later Cardiovascular Events. J Am Coll Cardiol. 2019;74(3):330–41. doi: 10.1016/j.jacc.2019.03.529 ; PubMed Central PMCID: PMC6764095. 31319915 PMC6764095

[25] Wadsworth SJ, Corley RP, Munoz E, Trubenstein BP, Knaap E, DeFries JC, et al. CATSLife: A Study of Lifespan Behavioral Development and Cognitive Functioning. Twin Res Hum Genet. 2019;22(6):695–706. Epub 20190924. doi: 10.1017/thg.2019.49 ; PubMed Central PMCID: PMC7487141. 31547893 PMC7487141

[26] Herink M, Ito MK. Medication Induced Changes in Lipid and Lipoproteins. In: Feingold KR, Anawalt B, Boyce A, Chrousos G, de Herder WW, Dhatariya K, et al., editors. Endotext. South Dartmouth (MA) 2000. 26561699

[27] Marshall AL, Miller YD, Burton NW, Brown WJ. Measuring total and domain-specific sitting: a study of reliability and validity. Med Sci Sports Exerc. 2010;42(6):1094–102. doi: 10.1249/MSS.0b013e3181c5ec18 . 19997030

[28] Hamilton CM, Strader LC, Pratt JG, Maiese D, Hendershot T, Kwok RK, et al. The PhenX Toolkit: get the most from your measures. Am J Epidemiol. 2011;174(3):253–60. Epub 20110711. doi: 10.1093/aje/kwr193 ; PubMed Central PMCID: PMC3141081. 21749974 PMC3141081

[29] Labouvie EW. Problem Behavior and Psychosocial Development; a Longitudinal Study of Youth. Journal of Studies on Alcohol. 1978;39(5):948–9. doi: 10.15288/jsa.1978.39.948

[30] Taylor-Piliae RE, Norton LC, Haskell WL, Mahbouda MH, Fair JM, Iribarren C, et al. Validation of a new brief physical activity survey among men and women aged 60–69 years. Am J Epidemiol. 2006;164(6):598–606. Epub 20060713. doi: 10.1093/aje/kwj248 . 16840522

[31] Trubenstein BP. The Importance of Place in Adults Approaching Midlife: University of California, Riverside; 2020.

[32] Ainsworth BE, Haskell WL, Herrmann SD, Meckes N, Bassett DR, Jr., Tudor-Locke C, et al. 2011 Compendium of Physical Activities: a second update of codes and MET values. Med Sci Sports Exerc. 2011;43(8):1575–81. doi: 10.1249/MSS.0b013e31821ece12 . 21681120

[33] Mendes MA, da Silva I, Ramires V, Reichert F, Martins R, Ferreira R, et al. Metabolic equivalent of task (METs) thresholds as an indicator of physical activity intensity. PLoS One. 2018;13(7):e0200701. Epub 20180719. doi: 10.1371/journal.pone.0200701 ; PubMed Central PMCID: PMC6053180. 30024953 PMC6053180

[34] Yi SW, Yi JJ, Ohrr H. Total cholesterol and all-cause mortality by sex and age: a prospective cohort study among 12.8 million adults. Sci Rep. 2019;9(1):1596. Epub 20190207. doi: 10.1038/s41598-018-38461-y ; PubMed Central PMCID: PMC6367420. 30733566 PMC6367420

[35] Lewis DA, Kamon E, Hodgson JL. Physiological differences between genders. Implications for sports conditioning. Sports Med. 1986;3(5):357–69. doi: 10.2165/00007256-198603050-00005 . 3529284

[36] Kuan PX, Ho HL, Shuhaili MS, Siti AA, Gudum HR. Gender differences in body mass index, body weight perception and weight loss strategies among undergraduates in Universiti Malaysia Sarawak. Malays J Nutr. 2011;17(1):67–75. . 22135866

[37] Downer B, Estus S, Katsumata Y, Fardo DW. Longitudinal trajectories of cholesterol from midlife through late life according to apolipoprotein E allele status. Int J Environ Res Public Health. 2014;11(10):10663–93. Epub 20141016. doi: 10.3390/ijerph111010663 ; PubMed Central PMCID: PMC4211000. 25325355 PMC4211000

[38] Wang I, Kapellush J, Hou S, Rahman M, Li X, Ritchie D. Trends in TC/HDL and LDL/HDL Ratios across the Age Span: Data from the 2007–2018 National Health and Nutrition Examination Survey (NHANES). Asian Journal of Complementary and Alternative Medicine. 2021;9(1):6–15. Epub 01/22/2021.

[39] Centers for Disease Control and Prevention NCfHS. National Health Interview Survey (NHIS): Diet and nutrition questionnaire. Questions NAC.040_00.00 (question 1), NAC.070_00.00 (question 2), NAC.090_00.00 (question 3), NAC.100_00.00 (question 4), NAC.110_00.00 (question 5), NAC.120_00.00 (question 6), NAC.130_00.00 (question 7), NAC.131_00.00 (question 8), and NAC.132_00.00 (question 9). 2005.

[40] Institute NC. Five-factor screener: National Health Interview Survey (NHIS) diet and nutrition. NAC.010-NAC.138. 2005.

[41] Pinheiro J. nlme: Linear and Nonlinear Mixed Effects Models. In: Bates D, editor. 2022.

[42] Reynolds CA, Smolen A, Corley RP, Munoz E, Friedman NP, Rhee SH, et al. APOE effects on cognition from childhood to adolescence. Neurobiol Aging. 2019;84:239 e1- e8. Epub 20190418. doi: 10.1016/j.neurobiolaging.2019.04.011 ; PubMed Central PMCID: PMC6800620. 31126628 PMC6800620

[43] Carlin JB, Gurrin LC, Sterne JA, Morley R, Dwyer T. Regression models for twin studies: a critical review. Int J Epidemiol. 2005;34(5):1089–99. Epub 20050808. doi: 10.1093/ije/dyi153 . 16087687

[44] Parker D, Sloane R, Pieper CF, Hall KS, Kraus VB, Kraus WE, et al. Age-Related Adverse Inflammatory and Metabolic Changes Begin Early in Adulthood. J Gerontol A Biol Sci Med Sci. 2019;74(3):283–9. doi: 10.1093/gerona/gly121 ; PubMed Central PMCID: PMC6376106. 29985987 PMC6376106

[45] Montonen J, Boeing H, Schleicher E, Fritsche A, Pischon T. Association of changes in body mass index during earlier adulthood and later adulthood with circulating obesity biomarker concentrations in middle-aged men and women. Diabetologia. 2011;54(7):1676–83. Epub 20110406. doi: 10.1007/s00125-011-2124-6 . 21468642

[46] Pletcher MJ, Bibbins-Domingo K, Liu K, Sidney S, Lin F, Vittinghoff E, et al. Nonoptimal lipids commonly present in young adults and coronary calcium later in life: the CARDIA (Coronary Artery Risk Development in Young Adults) study. Ann Intern Med. 2010;153(3):137–46. doi: 10.7326/0003-4819-153-3-201008030-00004 ; PubMed Central PMCID: PMC3468943. 20679558 PMC3468943

[47] Maas AH, Appelman YE. Gender differences in coronary heart disease. Neth Heart J. 2010;18(12):598–602. doi: 10.1007/s12471-010-0841-y ; PubMed Central PMCID: PMC3018605. 21301622 PMC3018605

[48] Espinosa De Ycaza AE, Donegan D, Jensen MD. Long-term metabolic risk for the metabolically healthy overweight/obese phenotype. Int J Obes (Lond). 2018;42(3):302–9. Epub 20170925. doi: 10.1038/ijo.2017.233 ; PubMed Central PMCID: PMC5867190. 29064474 PMC5867190

[49] Opio J, Croker E, Odongo GS, Attia J, Wynne K, McEvoy M. Metabolically healthy overweight/obesity are associated with increased risk of cardiovascular disease in adults, even in the absence of metabolic risk factors: A systematic review and meta-analysis of prospective cohort studies. Obes Rev. 2020;21(12):e13127. Epub 20200901. doi: 10.1111/obr.13127 . 32869512

[50] Shai I, Rimm EB, Hankinson SE, Curhan G, Manson JE, Rifai N, et al. Multivariate assessment of lipid parameters as predictors of coronary heart disease among postmenopausal women: potential implications for clinical guidelines. Circulation. 2004;110(18):2824–30. Epub 20041018. doi: 10.1161/01.CIR.0000146339.57154.9B . 15492318

[51] Luna MG, Pahlen S, Corley RP, Wadsworth SJ, Reynolds CA. Frailty and Processing Speed Performance at the Cusp of Midlife in CATSLife. J Gerontol B Psychol Sci Soc Sci. 2023. Epub 20230722. doi: 10.1093/geronb/gbad102 . 37480567 PMC10645312

[52] Piirtola M, Kaprio J, Svedberg P, Silventoinen K, Ropponen A. Associations of sitting time with leisure-time physical inactivity, education, and body mass index change. Scand J Med Sci Sports. 2020;30(2):322–31. Epub 20191024. doi: 10.1111/sms.13575 . 31605629

[53] Rottensteiner M, Leskinen T, Jarvela-Reijonen E, Vaisanen K, Aaltonen S, Kaprio J, et al. Leisure-time physical activity and intra-abdominal fat in young adulthood: A monozygotic co-twin control study. Obesity (Silver Spring). 2016;24(5):1185–91. doi: 10.1002/oby.21465 . 27112070

