# Supplementary material for: A twin-driven analysis on early aging biomarkers and associations with sitting-time and physical activity
Source: PLoS One. 2024 Sep 11;19(9):e0308660. doi: 10.1371/journal.pone.0308660 (PMC11389938; doi:10.1371/journal.pone.0308660)
Supplement: S1 Appendix — Methodology and process of calculating sitting time and exercise based on CATSLife participant self-report responses. (PDF) [file pone.0308660.s001.pdf]

# S1 Appendix
## Sitting Time Calculation

Total sitting time for weekdays was calculated by taking the total estimated sitting in all different categories reported during a typical weekday and then multiplying by five. Total sitting time for weekends was calculated by taking the total estimated sitting in all different categories during a typical weekend day and then multiplying by two. The sitting total on the five weekdays was added to the two weekend days to form a total sitting per week and then checked for any discrepancies. In 91 individuals an overlap between responses was evident where sitting time exceeded normal waking hours and adjustments in sitting hours were performed. Most individuals with discrepancies reported overlap in sitting time with their weekday work and weekday computer sitting hours, therefore, only one was counted. Another common instance of overlap was reporting the same high number of hours between time sitting at a computer, watching television, and other leisure time that amounted to an unreasonably high number of hours each weekday or weekend given the reported number of hours worked in a typical week. For example, if an individual reported working an average of 40 hours per week and listed eight hours of sitting per weekday while at work, but also eight hours of sitting per weekday at the computer and additional hours of sitting in another category per weekday the sitting at the computer was considered to be overlapped with the sitting at work hours. This overlap was adjusted to represent sitting throughout the workday as well as in leisure time. The maximum number of hours sitting per week was set at 112 given the time spent sleeping as well as the minimum amount of time needed to perform basic daily tasks that are not completed while sitting. Time spent sleeping and performing basic daily tasks that require standing was estimated to be around eight hours per day (i.e. sleeping between 6-8 hours, daily standing tasks between 0-2 hours). Examples of other non-sitting daily tasks that were considered were activities such as self-hygiene care, preparing or walking to get food, walking within the house, and walking to the vehicle or to one's desk.

## MET Minutes Per Week Calculation

Participants were asked to respond with a range of time that they spent doing the following activities each week (i.e., None, 1 hour or less, 2-3 hours, 4-5 hours, 6-7 hours, 8 or more hours a week): *taking part in an organized sport or recreation program, working out as a part of a personal exercise program*, *playing pickup games like basketball, touch football, etc.*, and *practicing different physical activities*. Responses were recoded to hours per week where the midpoint was taken for responses with hour ranges, i.e., 0=None, 1=1 hour or less, 2.5=2-3 hours, 4.5=4-5 hours, 6.5= 6-7 hours, 8=8 or more hours a week. Respondents were asked to provide short answer responses to three of the items describing the type or types of organized sport(s), exercise program(s), and physical activities they perform if they endorsed performing such activities at least 1 or more hours per week.

MET scores were assigned to these short-answer responses as previously mentioned within the methods. These MET scores were then checked for reliability between rater entries with reliability correlations of 0.79 for organized sports, 0.75 for exercise programs, and 0.77 for physical activities. A consensus MET rating was generated where disagreements were observed by four individuals not involved in original ratings (including co-authors SP and CR). Correlations between raters and consensus values were 0.83, 0.88, and 0.87 respectively, and for organized sports, exercise programs, and practicing physical activities. Answers to the hours of engagement in pickup sports did not include an open-ended response option, and any hours reported were added to the hours reported for organized sports. Of the participants reporting 1 or more hour of pick-up sports, 65% reported codable open-ended responses to organized sports.

To calculate the moderate MET minutes per week and vigorous MET minutes per week for each participant, the MET score of the activity was multiplied by the minutes per week value of each respective physical activity response derived from the hours per week value. In the event of multiple activities with different MET scores all classified as either vigorous or moderate, the mean was used in the instance of two scores present, and the geometric mean of the MET scores was used if three or more scores were present to account for possible large differences between MET scores. In the event of a participant having one or multiple MET scores belonging to both moderate and vigorous activity, the proportional time amount was calculated based on the total MET minutes. For example, if a participant noted 6.5 hours (6-7 hours a week) of working out as a part of an exercise program and gave short answer responses of jogging (vigorous MET score of 7.0), walking (moderate MET score of 3.5), and weightlifting (vigorous MET score of 6.0) a geometric mean of 5.28 was used to multiply by 390 minutes a week (6.5 hours multiplied by 60 minutes). The resulting 2059.2 MET mins per week were proportionally split up into 1/3 moderate MET minutes per week (686.093 mMETs) and 2/3 vigorous MET minutes per week (1372.1867 vMETs). In the uncommon event (18% of cases) that no short response answer was provided, an average moderate MET score of 4 was assigned to the calculation for MET minutes per week. To account for the overlap in time reported doing exercise as well as account for the under-reporting amount of daily physical activity, adjustments

were completed. Overlap was considered in approximately 15% of the cases and examples of such adjustments as well as other special instances can be found in Supplementary Figure 1. Overlap was considered and resulted in a reduction in MET minutes based on the following conditions:

- An overlap in short answers existed between activities (for example, if a respondent reported running for both exercise and physical activity responses)
- Clear overlap existed between activities based on time responses (for example, if a respondent reported 2-3 hours for each activity without distinctive differences in their short answers)
- Exercise hours were not possible given the time reported sitting by the respondent

Participants were also asked a separate close-ended question to mark the response best describing how they spent their leisure time during most of the last year. This question was leveraged with responses that were already given regarding the previously mentioned leisure activity questionnaire. If no leisure activity was previously reported, but a participant reported on average three times per week engaging in moderate activity, 240 moderate MET minutes were added. If the respondent answered that they engage in heavy physical activity at least three times per week, 540 vigorous MET minutes were added. If no previous activity was reported, but the respondent answered that they engaged in a regular physical fitness program for at least an hour or more on a daily or almost daily basis, 1800 MET minutes were added to their MET minutes per week.

Finally, participants were also asked to choose one of five options to best describe the kinds of physical activity they performed while on the job as well as give a short answer response of their occupation, if applicable. The question also denoted that if the respondent was not gainfully employed outside the home, but work around the home regularly, to consider that activity when answering the question. A response of spending most of the workday walking or using hands that required moderate exertion resulted in an addition of 240 moderate MET minutes. A response of spending most of the day lifting or carrying heavy objects resulted in an addition of 540 vigorous MET minutes. A response indicating the most demanding work reporting spending most of the day doing hard physical labor with only short breaks resulted in an addition of 1800 moderate or vigorous MET minutes based on the current job MET consensus assignment.
