# Supplementary material for: A twin-driven analysis on early aging biomarkers and associations with sitting-time and physical activity
Source: PLoS One. 2024 Sep 11;19(9):e0308660. doi: 10.1371/journal.pone.0308660 (PMC11389938; doi:10.1371/journal.pone.0308660)
Supplement: S1 Table — Table of medication families and specific medications that, if a participant reported taking, they were excluded from the biomarker analysis due to contraindications supported by the literature. (PDF) [file pone.0308660.s002.pdf]

S1 Table

**Amiodarone**	Pacerone	Cordarone	Nexterone		
**Beta Blockers (older)**	Propranolol	Inderal	Innopran XL	Atenolol	Tenormin
	Metoprolol	Kapspargo Sprinkle	Lopressor	Toprol-XL	
**Loop diuretics**	Bumetanide	Bumex	Ethancrynic Acid	Edecrin	Furosemide
	Lasix	Torsemide	Soaanz	Demadex	
**Thiazide diuretics**	Chlorothiazide	Chlorothalidone	Hydrocholorothiazide	Indapamide	Metolazone
	Diuril	Hygroton	Thalitone	Microzide	Lozol
	Chlorthalid	Zaroxolyn			
**SGLT2 inhibitors**	Canagliflozin	Dapagliflozin	Empagliflozin	Metformin	Jardiance
	Glumetza	Invokana	Farxiga		
**Estrogen**	Estradiol	Premarin	Vagifem	Vivelle	Premarin
	Estrace	Estratab			
**Progestin**	Aygestin	Camila	Crinone	Errin	Menace
	Norethindrone	Nexplanon	Etonogestrel	Mirena	Levonorgestrel
**Select Estrogen Receptor Modulators**	Tamoxifen	Soltamox	Raloxifene	Evista	Duavee
	Toremifene	Fareston	Bazedoxifene	Ospemifene	Osphena
	Clomiphene				
**Danazol**	Danocrine				
**Anabolic steroids**	Nandrolone	Oxandrolone	Oxymetholone	Stanozolol	Trenbolone Acetate
	Methandienone	Dianabol	Boldenone undecylenate	Equipoise	Mibolerone
	Testex	Depotest	Antro-Esto	Trenbolone Acetate	Finajet
	Fluoxymesterone	Halotestin	Cheque	Stanozolol	Mesterolone
	Testim	AndroGel	Provision	Winstrol	Testosterone
	Fortesta				
**Corticosteroids**	Aristocort	Bubbli-Pred	Celestone	Cortone Acetate	Cotolone
	Entocort EC	Florinef Acetate	Medrol	Methylpred-DP	Prednicot
	Decadron	Deltasone	Praline	Cortisone	Dexamethasone Intensol
	Hydrocortisone	Emflaza	Prednisone		
**Protease inhibitors**	Indinavir sulfate	Tipranavir	Squinavir mesylate	Fosamprenavir	Darunavir
	Norvir	Invirase	Aptivus	Crixivan	Viracept
	Cobicistat	Darunavir	Lopinavir	Ritonavir	Nelfinavir Mesylate
	Atazanavir	Reyataz	Kaletra	Prezcobix	Prezista
	Evotaz	Lexiva	Atazanavir		
**Direct Acting Antivirals**	Harvoni	Ledipasvir	Sofosbuvir	Zepatier	Elbasvir
	Voxilaprevir	Mavyret	Glecaprevir	Pibrentasvir	Grazoprevir
	Epclusa	Velpatasvir	Vosevi		
**Cyclosporine**	Tacrolimus	Sandimmune	Prograf	Protopic	
**First Generation antipsychotics**	Chlorpromazine	Fluphenazine	Perphenazine	Procholorperazine	Thioridazine
	Moban	Adasuve	Stelazine	Navane	Mellaril
	Trivial	Triptafen	Modecate	Moditen	RhoFluphenazine
	Procomp	Haldol	Loxitane	Thiothixene	Trifluoperazine haloperidol
	Stemetil	Buccastem	Thorazine	Largactil	Loxapine
	Trillion	Prolixin	Molindone	Etrafon	Compro
**Second Generation antipsychotics**	Abilify	Aripiprazole	Aristada	Asenapine	Cariprazine
	Iloperidone	Invega	Latuda	Lurasidone	Lybalvi
	Pimavanserin	Quetiapine	Risperdal	Risperidone	Saphris

	Ziprasidone	Zyprexa	Clozapine	Clozaril	Nuplazid
	Olanzapine	Secuado	Seroquel	Fanapt	Paliperidone
	Versacloz	Geodon	Perseris	Vraylar	
**Anticonvulsants**	Phenytoin	Oxcarbazepine	Dilantin	Trileptal	
**Retinoids**	Tretinoin	Absorica	Isotretinoin	Accutane	Arazlo
	Differin	Adapalene	Fabior	Myorisan	Panretin
	Targretin	Bexarotene	Tazorac	Zenatane	Aklief
	Tazarotene	Atralin	Renova	Retin-A	Bexarotene
	Targretin	Avita	Soriatane	Retinol	Claravis
	Acitretin				
**Growth Hormone**	Genotropin	Humatrope	Norditropin	Nutropin	Serostim
	Zomacton	Somatropin			
**Cholesterol Meds/Others**	Statins	Spironolactone			
