# Supplementary material for: A twin-driven analysis on early aging biomarkers and associations with sitting-time and physical activity
Source: PLoS One. 2024 Sep 11;19(9):e0308660. doi: 10.1371/journal.pone.0308660 (PMC11389938; doi:10.1371/journal.pone.0308660)
Supplement: S2 Table — Co-twin control change in -2 log likelihood fit statistics based on removal of specific effects or interactions. (PDF) [file pone.0308660.s003.pdf]

S2 Table

TC/HDL Ratio & Sitting	-2 Log Likelihood	Δ chi-squared	DF	p-value
Base Model	885.2	.	.	.
Within Pair – no Zygosity Interaction	888.7	3.5	1	0.061
Within Pair Effect Removed	893.8	8.6	2	0.014*
Between Pair – no Zygosity Interaction	885.5	0.3	1	0.584
Between Pair Effect Removed	885.8	0.6	2	0.741
Zygosity Interactions Removed from Both	889.0	3.8	2	0.150
TC/HDL Ratio & Vigorous Physical Activity	-2 Log Likelihood	Δ chi-squared	DF	p-value
Base Model	886.6	.	.	.
Within Pair – no Zygosity Interaction	886.7	0.1	1	0.752
Within Pair Effect Removed	893.8	7.2	2	0.027*
Between Pair – no Zygosity Interaction	886.6	0	1	.
Between Pair Effect Removed	887.2	0.6	2	0.741
Zygosity Interactions Removed from Both	886.7	0.1	2	0.951
BMI & Vigorous Physical Activity	-2 Log Likelihood	Δ chi-squared	DF	p-value
Base Model	2323.5	.	.	.
Within Pair – no Zygosity Interaction	2323.5	0	1	.
Within Pair Effect Removed	2323.8	0.3	2	0.861
Between Pair – no Zygosity Interaction	2323.6	0.1	1	0.752
Between Pair Effect Removed	2330.8	7.3	2	0.026*
Zygosity Interactions Removed from Both	2323.6	0.1	2	0.951

Notes: DF = degrees of freedom.
* = significant at $p < 0.05$.
