# Supplementary material for: A twin-driven analysis on early aging biomarkers and associations with sitting-time and physical activity
Source: PLoS One. 2024 Sep 11;19(9):e0308660. doi: 10.1371/journal.pone.0308660 (PMC11389938; doi:10.1371/journal.pone.0308660)
Supplement: S3 Table — Results of LME models including the variable of Age2. Results indicate no significances for Age2. (PDF) [file pone.0308660.s004.pdf]

S3 Table - Supplementary Table 3. LME Parameters for TC/HDL and BMI – Age, Age^2 and Sex

TC/HDL Ratio	B	SE	DF	p-value	B	SE	DF	p-value
Intercept	2.919	0.119	603	<.001*	2.975	0.127	603	<.001*
Age	0.029	0.006	331	<.001*	0.035	0.008	330	<.001*
Age^2	---	---	---	---	-0.002	0.001	330	.211
Male	0.660	0.059	331	<.001*	0.659	0.059	330	<.001*
White	-0.040	0.138	331	.771	-0.039	0.138	330	.778
Non-Hispanic	0.062	0.145	331	.670	0.045	0.146	330	.776

BMI	B	SE	DF	p-value	B	SE	DF	p-value
Intercept	26.806	0.818	611	<.001*	26.542	0.870	611	<.001*
Age	0.087	0.042	340	.040*	0.060	0.052	339	.256
Age^2	---	---	---	---	0.008	0.009	339	.383
Male	1.121	0.040	340	.006*	1.122	0.402	339	.006*
White	-1.110	0.928	340	.232	-1.116	0.927	339	.230
Non-Hispanic	0.503	0.987	340	.611	0.595	0.991	339	.549

Notes: DF = degrees of freedom.
* = significant at $p < 0.05$.
