# Supplementary material for: A twin-driven analysis on early aging biomarkers and associations with sitting-time and physical activity
Source: PLoS One. 2024 Sep 11;19(9):e0308660. doi: 10.1371/journal.pone.0308660 (PMC11389938; doi:10.1371/journal.pone.0308660)
Supplement: S4 Table — Results of LME models including behavioral interactions and interactions with age. Results indicate no significances for these interactions. (PDF) [file pone.0308660.s005.pdf]

S4 Table - Supplementary Table 4. LME Parameters for TC/HDL and BMI – Sitting and Vigorous Physical Activity Interactions

TC/HDL Ratio	B	SE	DF	p-value	B	SE	DF	p-value
Intercept	3.026	0.125	600	<.001*	3.026	0.125	600	<.001*
mMETs	-0.025	0.018	310	.167	-0.026	0.018	308	.163
vMETs	-0.036	0.013	310	.007*	-0.039	0.014	308	.004*
Sitting	0.003	0.001	310	.018*	0.004	0.002	308	.023*
FruitVegs	-0.015	0.009	310	.092	-0.015	0.009	308	.091
Age	0.027	0.007	310	<.001*	0.028	0.008	308	<.001*
Male	0.627	0.065	310	<.001*	0.627	0.065	308	<.001*
White	-0.044	0.139	310	.753	-0.044	0.139	308	.752
Non-Hispanic	0.075	0.147	310	.612	0.078	0.147	308	.597
Age*vMETs	0.0001	0.000	310	.585	-0.0004	0.004	308	.902
Age*Sitting	0.0006	0.003	310	.844	0.0003	0.000	308	.366
vMETs*Sitting	---	---	---	---	-0.0004	0.001	308	.520
vMETs*Sitting*Age	---	---	---	---	-0.0001	0.000	308	.414

BMI	B	SE	DF	p-value	B	SE	DF	p-value
Intercept	27.824	0.858	608	<.001*	27.844	0.856	608	<.001*
mMETs	-0.200	0.127	318	.101	-0.194	0.122	316	.113
vMETs	-0.307	0.088	318	.001*	-0.339	0.090	316	<.001*
Sitting	0.022	0.009	318	.010*	0.032	0.011	316	.002*
FruitVegs	-0.132	0.059	318	.026*	-0.131	0.059	316	.028*
Age	0.091	0.051	318	.075	0.095	0.051	316	.062
Male	0.761	0.438	318	.083	0.769	0.437	316	.080
White	-0.869	0.932	318	.352	-0.881	0.930	316	.344
Non-Hispanic	0.267	1.004	318	.791	0.267	1.002	316	.790
Age*vMETs	-0.003	0.019	318	.869	-0.009	0.020	316	.653
Age*Sitting	0.002	0.002	318	.302	0.002	0.002	316	.258
vMETs*Sitting	---	---	---	---	-0.007	0.004	316	.098
vMETs*Sitting*Age	---	---	---	---	-0.0005	0.000	316	.585

Notes: DF = degrees of freedom.
* = significant at $p < 0.05$.
