# Supplementary figures and images for: A twin-driven analysis on early aging biomarkers and associations with sitting-time and physical activity
Source: PLoS One. 2024 Sep 11;19(9):e0308660. doi: 10.1371/journal.pone.0308660 (PMC11389938; doi:10.1371/journal.pone.0308660)

S1 Fig

Supplementary Figure 1 - MET mins Adjustments

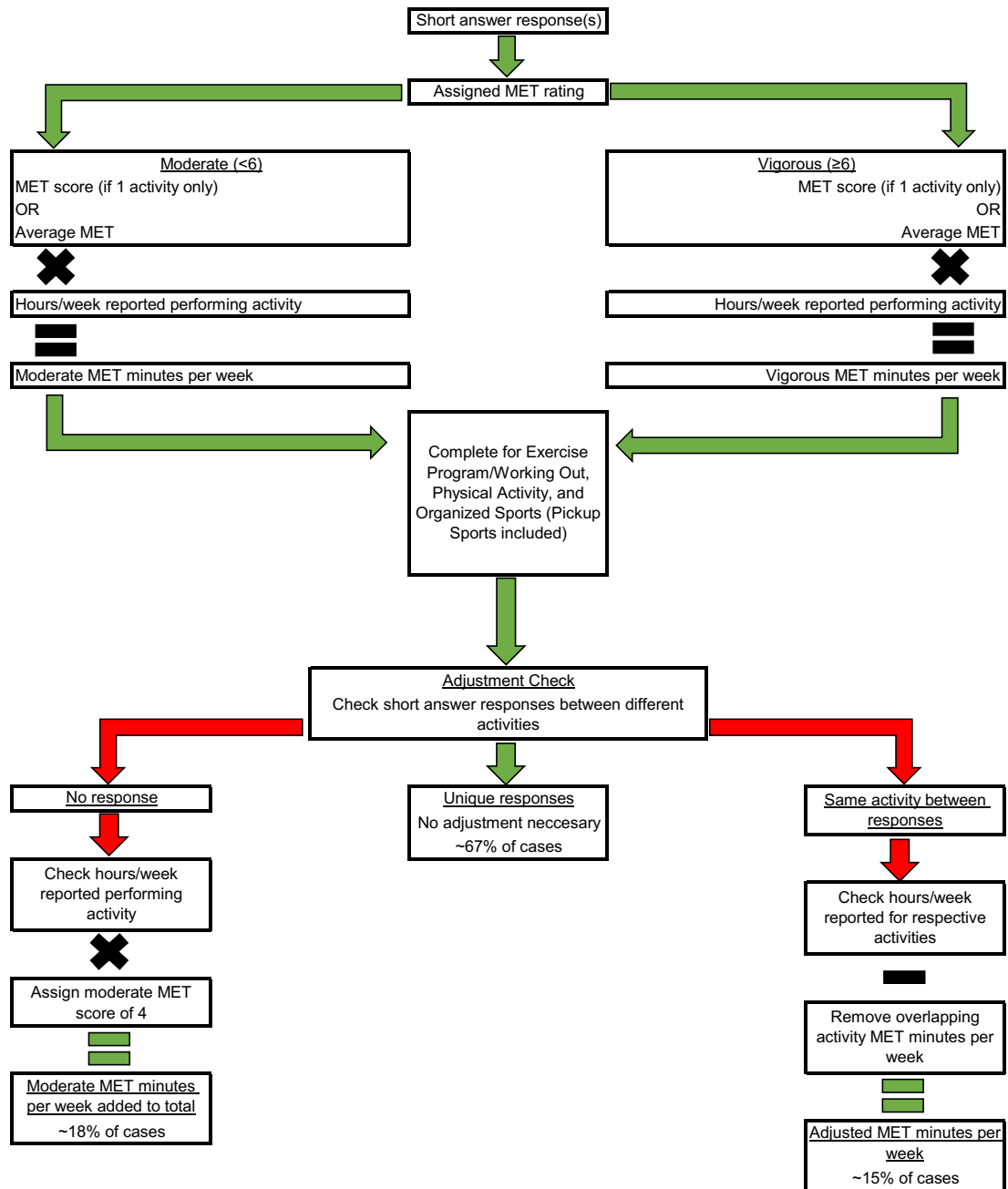

Supplement: S1 Fig — Visual representation of the adjustment process for calculating moderate and vigorous MET minutes per week for participants. (PDF) [file pone.0308660.s006.pdf]
